# Automation of Language Sample Analysis

**DOI:** 10.1044/2023_JSLHR-22-00642

**Published:** 2023-06-22

**Authors:** Houjun Liu, Brian MacWhinney, Davida Fromm, Alyssa Lanzi

**Affiliations:** a The Nueva School, San Mateo, CA; b Department of Psychology, Carnegie Mellon University, Pittsburgh, PA; c Communication Sciences and Disorders Department, University of Delaware, Newark

## Abstract

**Purpose:**

A major barrier to the wider use of language sample analysis (LSA) is the fact that transcription is very time intensive. Methods that can reduce the required time and effort could help in promoting the use of LSA for clinical practice and research.

**Method:**

This article describes an automated pipeline, called Batchalign, that takes raw audio and creates full transcripts in Codes for the Human Analysis of Talk (CHAT) transcription format, complete with utterance- and word-level time alignments and morphosyntactic analysis. The pipeline only requires major human intervention for final checking. It combines a series of existing tools with additional novel reformatting processes. The steps in the pipeline are (a) automatic speech recognition, (b) utterance tokenization, (c) automatic corrections, (d) speaker ID assignment, (e) forced alignment, (f) user adjustments, and (g) automatic morphosyntactic and profiling analyses.

**Results:**

For work with recordings from adults with language disorders, six major results were obtained: (a) The word error rate was between 2.4% for controls and 3.4% for patients, (b) utterance tokenization accuracy was at the level reported for speakers without language disorders, (c) word-level diarization accuracy was at 93% for control participants and 83% for participants with language disorders, (d) utterance-level diarization accuracy based on word-level diarization was high, (e) adherence to CHAT format was fully accurate, and (f) human transcriber time was reduced by up to 75%.

**Conclusion:**

The pipeline dramatically shortens the time gap between data collection and data analysis and provides an output superior to that typically generated by human transcribers.

Here, we present the results of application of an automated pipeline that creates transcripts in the Codes for the Human Analysis of Talk (CHAT) transcription format (https://talkbank.org/manuals/CHAT.pdf) from raw audio input. Use of the CHAT format is required for language samples to be added to TalkBank databases such as the Child Language Data Exchange System (for child language), FluencyBank (for stuttering), AphasiaBank (for aphasia), TBIBank (for traumatic brain injury [TBI]), PhonBank (for phonological development), BilingBank (for bilingualism), RHDBank (for right hemisphere disorders [RHDs]), ASDBank (for autism), and several others. Data from these banks have been used in over 9,000 publications, as reported through Google Scholar. Use of TalkBank data and programs forms a core part of the curriculum in speech and communication science in over 100 universities, and these methods are used in 24 countries to guide clinical practice. Because TalkBank repositories use the single common CHAT format, all the data in these corpora can be analyzed consistently using the Computerized Language Analysis (CLAN) programs (https://dali.talkbank.org) for corpus analysis and language profiling; the TalkBankDB database system (https://talkbank.org/DB), which provides an API (application programming interface) linking to R for statistical analysis; and the new Collaborative Commentary system (https://talkbank.org/CC), which facilitates transcript coding and analysis by groups of students, clinicians, and researchers. There are other popular methods for producing transcripts from raw audio, such as EUDICO Linguistic Annotator (ELAN; Wittenburg et al., 2006), Systematic Analysis of Language Transcripts (SALT; J. Miller & Chapman, 1983), and Sampling Utterances and Grammatical Analysis Revised (SUGAR; Pavelko & Owens, 2017). However, these other methods do not produce CHAT transcripts and therefore cannot be analyzed by these TalkBank systems for corpus analysis, language profiling, and collaborative commentary. Moreover, inclusion of data into TalkBank for open data sharing (Lin et al., 2020; Wilkinson et al., 2016) requires that the transcripts be in CHAT format.

Prior to the introduction of this pipeline, spoken language data contributed to TalkBank repositories had to be manually transcribed and coded in accord with CHAT format. In the 1970s and 1980s, transcribers often relied on a foot pedal to control the movement of an audiotape on a recording machine. Later, transcribers used personal computers to transcribe from digital audio. It is widely recognized that the tedious process of hand transcription creates a major barrier to the use of language sample analysis (LSA) in clinical practice (Bernstein-Ratner & MacWhinney, 2016). Even for nonclinical speech, the ratio of transcription time to recording time is 11:1 on average (Novotney & Callison-Burch, 2010). For clinical samples, that ratio can be much larger, particularly if it includes annotation of retraces, pauses, errors, and nonstandard phonology.

The TalkBank project has developed several methods to facilitate human transcription. One method, called Walker Controller, echoes the actions of the foot pedal for rewinding and advancing with keyboard function keys. Another, more time-consuming method, called Sonic CHAT, uses delineation of areas in a waveform display window for highly accurate alignment of transcripts to segments of the audio. A third method, called Transcriber Mode, allows the user to break up the audio into bulleted segments by pressing the space bar at the end of each utterance. After that, the user can return to each bulleted segment to replay and transcribe. Transcriber Mode is the most time-efficient method, but the utterance time values generated in this method do not take account of inter-utterance pauses and therefore do not accurately reflect utterance length. Moreover, none of these methods provides time alignment on the word level. For detailed phonological analysis, fluency analysis, and conversational sequencing analysis, it is important to have accurate marking of the beginning and end times for both words and utterances. This type of detailed time alignment or *diarization* can be performed manually in the Praat program (https://praat.org), but creating these alignments can be a tedious process.

To provide quicker and more accurate transcription and diarization, we created a pipeline, called Batchalign, that generates transcripts in CHAT format from raw audio samples. The pipeline performs automatic speech recognition (ASR), utterance tokenization, utterance- and word-level diarization, morphosyntactic coding, profiling, and fluency analysis with greatly reduced involvement from the researcher or clinician.

During its development, Batchalign was used to create word-level alignments for over 1,000 already-transcribed files in the AphasiaBank segment of TalkBank, as well as to generate new transcripts from 42 raw audio files in DementiaBank and 12 raw audio files in TBIBank. In this article, we describe the stages of the pipeline, its constituent tooling, and the use of human intervention to segment utterances, add annotations, and correct errors.

We formulate the research question here as how to best use speech technology to create an accurate and diarized CHAT transcript with maximal automation and minimal human input. The shape of CHAT format is specified in the CHAT manual at https://talkbank.org/manuals/CHAT.pdf, and accurate adherence to CHAT standards can be verified through use of the Chatter program available from https://talkbank.org/software/chatter.html. The transcript must include proper spelling of all spoken words, accurate combination of words into utterances, and assignment of utterances to the correct speaker. In addition, there must be proper marking of nonword forms, disfluencies, and repetitions. To allow for fluency analysis, there should be time values given for the beginning and end of each word and each utterance. There are six criteria for evaluating the success of this effort. The outcomes of interest are (a) word error rate (WER), (b) accurate utterance tokenization, (c) accurate word-level diarization, (d) accurate utterance-level diarization, (e) adherence to CHAT format, and (f) minimization of requirements for human clean-up. We evaluate these outcomes through application of the pipeline to a corpus from people with mild cognitive impairment (MCI) described below.

## Method

Batchalign combines two existing tools along with a variety of newly developed methods. Processing in the pipeline involves seven steps: (a) ASR, (b) utterance tokenization, (c) automatic corrections, (d) speaker ID assignment, (e) forced alignment (FA), (f) user adjustments, and (g) automatic morphosyntactic and profiling analyses. The design considerations and operations of each step are described in the following sections. The full pipeline, along with instructions for installation and usage, is available for download at https://github.com/talkbank/batchalign. It is freely available for Windows, Linux, and macOS without any password, and we encourage users to use it to transcribe and analyze recordings they have created. If data are being prepared for inclusion in TalkBank, we are happy to provide support for installation and use of the system.

### ASR

The first step in the pipeline involves sending an audio file over the web for processing by an ASR system. The output of this process is a diarized transcript. Diarization involves assigning utterances to specific speakers and marking the time values of the beginnings and ends of all utterances and words. Although the pipeline is agnostic regarding the ASR service being used, the current default version leverages the output of Rev AI (https://rev.ai), a commercial ASR service (C. Miller et al., 2022) with an online application programming interface (API). ASR is performed via the official REST API of Rev AI. When designing the pipeline, we evaluated six other commercial speech recognition systems, as well as the ESPNet research ASR system (Hayashi et al., 2020). The decision to use Rev AI was based on three considerations.

First, the downstream tasks in the pipeline—FA and morphology analysis—require that the transcript contain the exact words spoken to extract phoneme information, parts-of-speech, and other relevant word-level information. However, systems such as Amazon Transcribe output numbers in the form of Arabic numerals, rather than spoken language words. For lexical, phonological, and grammatical analyses, we needed the ASR system to provide accurate renditions of these word forms without postprocessing. Rev AI allows for this by making it possible for the user to turn off inverse text normalization that would convert *five dollars* to $5.

Second, a feature of most commercial ASR systems that makes them unsuitable for our task is the automatic removal of filled pauses, repetitions, and other verbal disfluencies (e.g., *you know*, *um*, *mhm*). Although this removal may be advantageous for many uses, it misrepresents the actual language being used and blocks the use of the data for fluency analysis. Fortunately, ASR through Rev AI includes an option for retaining these features.

Third, the Rev AI system has achieved important advances in accuracy based on the recent introduction of an end-to-end (E2E) system that leverages the availability of corpora for training a deep-learning model. The Rev AI system processes more than 15,000 hr of human-checked transcription each week across a wide variety of languages, building up a large database for training. C. Miller et al. (2022) present a fuller account of the details of the current Rev AI system and its use of human annotation to improve ASR performance. Jetté (2022) explains the computational construction of the new E2E system.

Upon completion, the ASR returns a time-aligned string of words spoken, a numerical tag for each predicted speaker, and grouping of words spoken by each speaker into time-aligned turns. Batchalign replaces these time values with more accurate values in Step 3. However, these initial time values help guide further steps in the pipeline.

### Utterance Tokenization

The second step in the processing pipeline involves segmenting the word stream into utterances. ASR systems, such as Rev AI, provide output as a string of words (Malik et al., 2021) grouped into turns based on speaker identity. However, like other LSA methods, the CHAT format and the CLAN programs are designed to work on utterances rather than turns. Furthermore, analysis of retracing or fluency measures, such as the mean length of utterance, requires that the words be assembled into properly delimited utterances (Brown, 1973). In addition, use of TalkBank's MOR/MEGRASP system (https://talkbank.org/manuals/mor.pdf) for automatic morphosyntactic analysis assumes that the transcript is composed of utterances. Therefore, the pipeline must include a system that accurately organizes words into utterances.

#### Conventional Utterance Segmentation

Speech utterance segmentation presents obvious parallels to traditional sentence segmentation. However, segmentation of talk data provides unique challenges (Fraser et al., 2015). We treated the problem of utterance segmentation as one of utterance tokenization followed by punctuation restoration. Conventional tokenization schemes tend to group coordinate clauses together, even when they are being produced as separate units in spoken language. For instance, consider the following output from ASR:

they get their father and the father climbs up the tree to try and get the cat

Conventional punctuation restoration approaches (Chen et al., 2021; Nagy et al., 2021) will produce this single utterance output:

They get their father and the father climbs up the tree to try and get the cat.

However, in accordance with the principles for utterance segmentation summarized in pages 60–72 of Chapter 9 of the CHAT manual (https://talkbank.org/manuals/CHAT.pdf), and in alignment with the concept of T-units (Foster et al., 2000), we would want this to be segmented into these two utterances:

they get their father.

and the father climbs up the tree to try and get the cat.

#### A Novel Utterance Tokenizer

To address this issue, we devised a novel segmentation model. Fortunately, the TalkBank corpora already contain many Gold Standard utterances that were segmented by hand in accordance with the required standards. Using these data, we trained a novel utterance segmentation model consistent with the existing literature (Wu et al., 2022) that treats text segmentation as a token-level sequence labeling punctuation restoration task.

The segmentation model was trained via Hugging Face (Wolf et al., 2020) with a BERT-base model (Devlin et al., 2018) using the entirety of the CHAT version of the MICASE (The Michigan Corpus of Academic Spoken English; Römer, 2019) corpus in TalkBank. MICASE (https://ca.talkbank.org/access/MICASE.html) includes transcribed data from 300 participants in a wide variety of interactions between students and faculty at the University of Michigan. It includes 165 transcripts with 1,634,035 words, along with 11.7 GB of audio. These interactions include advising, colloquia, thesis defense, interviews, lab meetings, office hours, seminars, service encounters, student presentations, study groups, and campus tours. The interactions have all been transcribed in CHAT with full adherence to CHAT transcription conventions. Each utterance is delimited by a period, question mark, or exclamation mark; the placement of these marks is therefore used to mark the ends of utterances.

The first step of utterance segmentation involves grapheme-level tokenization via the Hugging Face 4.23.1 Byte-Pair encoding tokenizer (Wolf et al., 2020). Each resulting token is assigned one of six labels by the model: If the grapheme to be labeled is predicted to be adjacent to the end of the utterance—that is, if a period, question mark, or exclamation mark follows it—it is assigned Label 2 for periods, Label 3 for question mark, or Label 4 for exclamation mark based on their punctuation. If a comma follows the token, it is assigned Label 5. Tokens whose first character is capitalized are assigned Label 1. Finally, if the token is not assigned a label based on the previous characteristics, it is assigned Label 0.

The second step, after obtaining the model prediction on graphemes, is to assemble passages based on the labeled graphemes with the predicted punctuation added. For instance, if a grapheme is predicted to be in Group 2, a period character is added to the end of the token during assembly.

The third and final step to obtain utterance tokenization is to pass the reassembled passage into the NLTK Punkt tokenizer (Bird et al., 2009), which will split the utterance based on the newly introduced punctuation. This results in data that are formatted in accordance with CHAT standards. Except for use of NLTK Punkt, all segments of this second step involve novel programming.

### Automatic Correction

The third step in the pipeline involves the use of novel transcript correction programs to reformat certain common words and codes in the output of the second step. These corrections are designed to maximize the accuracy of the programs for morphosyntactic analysis and language profiling used in the final step of the pipeline because those programs work best when words are spelled accurately and when disfluencies are marked properly.

First, the pipeline scans and replaces transcriptions of individual forms. It replaces filled pauses, such as *um*, with more distinctive forms such as *&-um*, thereby enabling downstream analysis and profiling programs to correctly tabulate disfluency behavior. It standardizes forms like *mm-hmm* and *mm-hum* that express agreement as the single form *mhm*. It uses CLAN's LOWCASE command and its large proper noun database to ensure that all proper nouns are capitalized, thereby facilitating morphological and syntactic analysis. The pipeline can also combine two or more words that function syntactically as a single unit into one word with one or more underscores. For example, *in between* is converted to *in_between* and *on account of* is converted to *on_account_of*. Finally, using CLAN's RETRACE program, the pipeline creates clear markings of word repetition by adding the marker [/] between repeated forms. As in the second step, all components of this step involve novel programming, although some of the pieces include modified versions of existing C++ code for CLAN commands.

At this point, the pipeline generates a draft CHAT file to be used by further steps in the system. The draft transcript contains annotated speaker tiers, speaker diarization, segmented utterances, and the corrected word forms mentioned above. Up to this point, all pipeline processing has been entirely automatic. The next section describes a role for brief human intervention to assign speaker IDs.

### Speaker ID Assignment

The fourth step in the pipeline involves human intervention to assign speaker ID codes. CHAT requires that each speaker be given a conversational role and a speaker ID. For example, the target child in studies of language development has the role *Target_Child* and uses *CHI* as the ID. In clinical interviews, the investigator or clinician has the role *Investigator* and the ID of *INV*. The output of ASR from Rev AI identifies speakers as Speaker0, Speaker1, Speaker2, and so forth. To assign the correct role and ID to each Rev AI code, the program presents the user with the longest example utterance from each speaker along with the time of that utterance. This allows the user to replay the segment to determine who is speaking, although this is sometimes clear just from reading the text. For example, it could be that Speaker1 is the child being recorded. In this case, the user enters the three-letter code *CHI* for the child and the role *Target_Child*. For transcripts with four or fewer speakers, this process takes approximately 2 min to complete.

### FA

The fifth step in the pipeline involves passing the transcript to a program for FA. FA is a process that associates each utterance and each word with a beginning and ending time. In CHAT format, these times are given in milliseconds, starting from the beginning of the recording. For this process, we elected to use the Montreal Forced Aligner (MFA; McAuliffe et al., 2017) with speaker adaptation and grapheme-to-phoneme (G2P) dictionary generation. Mahr et al. (2021) showed that, with child language samples, MFA performed the best of all standard FA tools, achieving 87% accuracy at the phoneme level. Diarization at this level is beyond what we are currently using for the TalkBank language sample analyses reported on here, but this level is available from MFA if needed. Mahr et al. (2021) attribute the superior performance of MFA to the relatively larger size of its training corpus and the use of speaker adaptation. They note that no aligners are currently trained with child language data, and they believe that the collection and use of child language training data could markedly improve current performance.

The lattice alignment scheme in Kaldi (Povey et al., 2011), on which the MFA is built, does not perform well with large intervals of data (beyond 1–2 min). Fortunately, MFA provides the ability to split audio into segments before supplying it to Kaldi—provided a Praat TextGrid with segmented transcripts and corresponding audio intervals is available in conjunction with the audio. These initial values can be derived from two possible sources. When using the pipeline with ASR, the time values for utterances provided by Rev AI can be used as provisional values. Alternatively, when processing transcripts with utterance-level time values already marked, one can use MFA directly by adding the *-prealigned* switch to the pipeline command. In both cases, CLAN's CHAT2PRAAT program extracts initial rough alignments from the source CHAT file, along with cleaned versions of the utterance, and places them into a TextGrid file for use by MFA. MFA will then replace the provisional alignments with final alignments.

The MFA segment of the pipeline uses the English (United States) ARPA G2P Model 2.0.0 to generate a dictionary from the graphemes of all the words in the transcript. It scans the entire input transcript and converts all graphemes to combinations of possible phonemes. These phoneme combinations are then used by the English (United States) ARPA acoustic Model v2_0_0 trained using the Librispeech corpus (Panayotov et al., 2015). After a series of trials, we found that using an alignment beam width of 100 words produces the most accurate alignment for the transcripts used here.

A list of time-annotated words is then returned by MFA in the format of a Praat TextGrid file. New utterance segmentations and word-level alignments are generated by matching the words inside the TextGrid file with the original input CHAT transcript—ensuring that disfluency annotations previously supplied are retained.

For each diarized speaker utterance in the input, the final CHAT file contains the main utterance tier with utterance-level alignment information in “bullets” at the end of each utterance with start and stop times in milliseconds. These bullets can be collapsed to increase readability or expanded to show the times. The pipeline inserts a %wor tier for each utterance with millisecond time-marking bullets after each word. For example, consider the following input:

*INV: + < &-um (.) how do you think your speech is these days? •52004_54174•

FA with MFA produces the following transcript:

*INV: + < &-um (.) how do you think your speech is these days? •52004_54174•

%wor: + < &-um (.) how •52004_52204• do •52204_52284• you •52284_52404• think •52404_52764• your •52764_52864• speech •52864_53434• is •53474_53554• these •53744_53914• days •54024_54174•?

In the CLAN editor, the verbose time values can be reduced to a single bullet character by typing ESC-a, making the utterance appear in the following form:

*INV: + < &-um (.) how do you think your speech is these days? •

%wor: + < &-um (.) how • do • you • think • your • speech • is • these • days •?

### Human Correction

The sixth step in the pipeline involves human correction. Up to this point, processing of a 30-min recording takes 7 min or less. This is considerably faster than the 8 hr or more for a human transcriber to reach this same level. Moreover, the human transcriber would not have been able to mark time periods for words and utterances. However, there are several steps that must be completed before the transcript is ready for further analysis.

The first step involves running the CHECK program in the CLAN editor by typing ESC-L. CHECK requires that the time values on the utterances all be sequential. In other words, an utterance that is later in the transcript cannot have a time value that is earlier than the preceding utterance. Occasionally, ASR assigns incorrect start times to utterances composed of single words such as *sure* or *yeah*. To correct this, one must open the bullet marks in the transcript using ESC-a to adjust the time values to be sequential. An effective way of dealing with this is to code such back-channel comments using the & = * code, as in this example:

*PAR: well, I was still recovering & = *INV:yeah from my stroke.

After fixing the problems with utterance times that were noted by CHECK, the ESC-8 continuous playback function can be used to play through the entire transcript. For a 30-min transcript, this will take approximately 1 hr, allowing for time for corrections during playback. A simple type of correction involves repairing misspellings or incorrect words. Such errors occur most frequently when ASR is not able to recognize an out-of-vocabulary proper noun. These errors can be corrected during the playback step. Another type of correction involves the addition of special marks for sentence termination. ASR ends each utterance with a period, and sometimes this needs to be changed to a question mark (?), an exclamation mark (!), or an incompletion mark (+…). During this process, it is also easy to correct any errors in speaker assignment made by Rev AI. CLAN provides a menu of shortcut keys that can switch assignment between speakers with a single shortcut such as command-2.

CLAN also provides a method for automatically joining or splitting utterances. Because utterance-level timecodes are linked to the word-level time codes, resegmenting utterance-level timecodes by hand requires tedious adjustment of the word-level timecodes. To avoid this problem, the user can mark places for resegmentation with the symbol &&& and then run CLAN's SEGMENT program to reformat automatically. After these corrections, the language sample is ready for analysis. There are two sets of facilities available in CLAN to enable further automatic analysis of the completed data: morphosyntactic analysis and profiling commands.

### Morphosyntactic Analysis

To generate automatic profile analyses, it is not enough to just have an accurate and diarized transcript. Profiling programs also require that the language be analyzed for its morphological and syntactic structure. CLAN provides a chain of analytic tools for this step in the analysis. The five components of this chain are MOR, PREPOST, POST, POSTMORTEM, and MEGRASP. MOR reads a set of lexical files to create a runtime lexicon based on left-associative grammar (Hausser, 1989). After MOR completes, there may be some ambiguities to be resolved and some patterns that require correction. PREPOST works to disambiguate forms such as nonverb ambiguities based on local context. Then POST uses part-of-speech sequence regularities to disambiguate between parts of speech. POSTMORTEM then corrects a few remaining structures. The result is an unambiguous analysis of the morphological components of all words on the %mor line. MEGRASP then uses the part-of-speech categories on the %mor line to create a dependency grammar analysis (Kübler et al., 2009) for each utterance. This analysis is placed on a %gra line, which users can double-click to invoke the GraphViz web service to display a fully connected graph of the structure with labeled nodes and labeled directed arcs. The chain runs automatically on any collection of files through the single command: mor *.cha. A full description of the operation and requirements of the chain is provided in the MOR manual at https://talkbank.org/manuals/mor.pdf.

### Profiling Commands

Once the %mor and %gra lines are completed and checked, transcripts can be analyzed using CLAN's language profiling analyses to compute a variety of clinically relevant indices along with comparisons to the database. These analyses include Developmental Sentence Score (DSS), Index of Productive Syntax (IPSyn), KIDEVAL, C-NNLA, C-QPA, EVAL, EVAL-D, and FluCalc. The DSS (Lee, 1974) and IPSyn (MacWhinney et al., 2020; Scarborough, 1990) are measures of the usage of morphosyntactic structures that were originally created for the study of child language. However, they have also proven useful for the study of speech from adults with language disorders. Trained users of DSS and IPSyn may require as much as an hour to create these profiles by hand (Overton & Wren, 2014), whereas they can be created in less than a minute using the computer versions.

EVAL (Forbes et al., 2012), C-NNLA (Fromm et al., 2020; Thompson et al., 1995), and C-QPA (Fromm et al., 2021; Rochon et al., 2000) are computerized versions of measures designed for analysis of language in aphasia. EVAL-D is a version of EVAL designed specifically for analysis of language in dementia. FluCalc (Bernstein-Ratner & MacWhinney, 2018) is designed to measure disfluencies, such as initial segment repetition, blocking, prolongation, pausing, word repetition, phrase repetition, and retracing. FluCalc uses the accurate word-level and utterance-level diarization produced by MFA to construct a full profile of fluency or disfluency in each participant. Details regarding the measures included in each of these profile analyses can be found in the CLAN manual at https://talkbank.org/manuals/CLAN.pdf.

For narrative and picture description tasks, CLAN's CoreLex program (Dalton et al., 2022) can measure the typicality of lexical use in describing the major elements of a picture or story. For example, when telling the Cinderella story, the program looks for mention of *slipper*, *pumpkin*, *stepmother*, and other core lexical items. The combination of EVAL-D, FluCalc, and CoreLex provides clinicians with a rich set of measures that can then be compared automatically against norms and benchmarks in the larger AphasiaBank or DementiaBank protocol databases in terms of means and standard deviations. For researchers building machine learning (ML) analyses (Luz et al., 2021), these profiling systems provide all the measures that have been shown to be important in diagnosing dementia with the exception of acoustic features.

A reference implementation of the pipeline used to obtain the results reported above is available to the public on the free Anaconda platform on all major operating systems. After installing a copy of Anaconda, users can follow the instructions available on the TalkBank GitHub page (https://github.com/TalkBank/batchalign) to obtain and use the pipeline.

## Results

We evaluate the success of the pipeline along six dimensions: (a) WER, (b) utterance tokenization accuracy, (c) word-level diarization accuracy, (d) utterance-level diarization accuracy, (e) adherence to CHAT format, and (f) minimization of requirements for human clean-up. Our analysis is based on the pipeline's processing of untranscribed audio from the Delaware corpus in DementiaBank (Lanzi, 2021) available from https://dementia.talkbank.org with doi:10.21415/Q0JX-5 W20. These recordings were collected over the Internet during 2021–2022 through Zoom from 38 individuals with MCI and 21 age- and education-matched healthy controls.

The language samples were elicited using the DementiaBank protocol, which is available from https://dementia.talkbank.org/protocol/. The tasks in this protocol include the Cookie Theft picture description (Goodglass et al., 2000), the Cat Rescue picture narrative (Nicholas & Brookshire, 1993), Rockwell's “Going and Coming” (Rockwell, 1947), the Cinderella picture book story retell (Grimes, 2005), how to make a peanut butter and jelly sandwich, and a personal narrative. The 38 MCI transcripts have 26,413 words spoken by the patients, and the 21 control transcripts have 20,379 words spoken by the control participants. The total duration of these recordings is just over 10 hr.

Diagnosis as MCI was based on results from the Montreal Cognitive Assessment (Nasreddine et al., 2005), the Boston Naming Test–Short Form (Kaplan et al., 2001), the Hopkins Verbal Learning Test–Revised (Benedict et al., 1998), and the Wechsler Memory Scale–Revised (Wechsler, 1987). Further details regarding inclusion/exclusion criteria, demographics, and evaluation criteria are given in Lanzi et al. (2023).

### WER

To evaluate the accuracy of ASR using Rev AI, we first examined the WER, which is defined as the sum of word insertions, deletions, and substitutions, divided by the total number of words. We took a random sample of 1% of the 26,413 words in the 38 MCI transcripts and a 1% random sample of the 20,379 words in the transcripts from the 21 healthy controls. WER was 3.6% for the MCI patients and 2.4% for the controls. Occasional ASR errors such as these can be fixed during the final playback process in the CLAN editor. Because this error rate is so low, there are minimal impacts of these few errors on the features that are involved in the next stage of utterance tokenization, thereby minimizing any possible compounding of error through the stages of the pipeline.

### Utterance Tokenization Accuracy

Next, we evaluated the accuracy of the system for utterance tokenization. We did this by comparing the first pass results from the pipeline with the human-corrected final transcripts from the Delaware corpus. We achieved an *F*_1_ score of 86.9% for MCI and 85.1% for the controls. Table 1 compares these results for the Batchalign pipeline with representative samples (Alam et al., 2020; Chen et al., 2021; Fraser et al., 2015; Shi et al., 2021) of state-of-the-art results from other tokenization systems that used formal speech data from TED Talks (Federico et al., 2012). Those results are given in the last four rows of Table 1. That table reports accuracy in terms of precision, recall, and *F*_1_ scores. *Precision* is defined as the number of true positives divided by the number of all positive results, including the false positives. *Recall* is defined as the number of true positives divided by the number of all cases that should be tagged as positive, including those that were incorrectly tagged as negative. *F*_1_ *scores* are defined as the harmonic mean of precision and accuracy. The *F*_1_ scores obtained by these other systems are quite comparable to those obtained by Batchalign, even though an analysis dealing with multiparty clinical interview data is inherently more challenging than one dealing with prepared monologue. Compared to the four models based on TED talk data, Batchalign achieves a higher recall and a lower precision because it is sometimes overly greedy in splitting utterances. These utterance splitting errors can be corrected by using the SEGMENT tool in CLAN, as described earlier.

**Table 1.** Precision, recall, and *F*_1_ values in percentage for sentence tokenization.

Model/corpus	Precision	Recall	*F*_1_
Batchalign-Delaware MCI	86.2	90.0	86.9
Batchalign-Delaware Controls	81.7	88.8	85.0
CRF (Fraser et al., 2015)	–	–	43.0
RoBERTa-base (Alam et al., 2020)	84.0	83.9	83.9
RoBERTa-large+SCL (Alam et al., 2020)	84.8	83.1	83.9
FT + POS + SBS (Shi et al., 2021)	82.9	85.7	84.3
ELECTRA-large+Disc-ST (Chen et al., 2021)	83.7	86.7	85.2

*Note.* Dashes indicate data not reported. *F*_1_ values = the harmonic mean of precision and recall; MCI = mild cognitive impairment; CRF = conditional random field; SCL = supervised contrastive learning; POS = part of speech; SBS = sequence boundary sampling; FT = Funnel Transformer; Disc-ST = discriminative self-training.

Table 1 also presents *F*_1_ scores obtained in the only other published study of tokenization of clinical samples from Fraser et al. (2015). That study used a conditional random field (CRF) method (Okazaki, 2007) to identify utterance boundaries. The input to the analysis was a hand-corrected transcription that was force aligned to the audio, providing lexical and prosodic information, along with inter-word time values. The CRF system was applied to aligned transcripts from 11 patients with semantic dementia (SD), 17 patients with progressive nonfluent aphasia (PNFA), and 23 age- and education-matched healthy controls. Neither SD nor PNFA is characterized by marked articulatory problems, but rather by problems with sentence formulation and lexical retrieval. Speech samples were elicited using retelling of the Cinderella story after paging through a wordless picture book, following the same method used in the AphasiaBank protocol (https://aphasia.talkbank.org/protocol/english/) and the DementiaBank protocol. The *F*_1_ scores from this analysis were .43 for SD, .47 for PNFA, and .51 for controls. Fraser et al. (2015) also report an *F*_1_ score of .57 for a similar-sized segment of the TDT4 Broadcast News corpus (catalog.ldc.upenn.edu/LDC2005S11). These *F*_1_ utterance segmentation scores are much lower than the *F*_1_ scores for Batchalign as shown in Table 1.

### Word-Level Diarization Accuracy

Mahr et al. (2021) explored the use of MFA for the task of child language alignment. We expanded upon the evaluation of Mahr et al. by comparing the performance of Batchalign on word-level alignment of audio from participants with MCI and controls (Lanzi, 2021). We sampled the performance of Batchalign automatically and evaluated it manually. We selected a 1% random sample of each group in the corpus, binned by cognitive impairment, with the number of words as described in the first column of Table 2. Based on preliminary trials, the performance of the aligner on mono- and multisyllabic words can vary significantly. Therefore, care was taken to also bin the monosyllabic data separately.

**Table 2.** Percentage of results annotated to be within alignment either fully or partially; confidence bands computed with *t* test at 95% confidence.

*N* words	Group	Syllable count	Fully or partially aligned
277	Control	Monosyllabic	90.61% ± 3.46%
278	Control	Multisyllabic	96.75% ± 2.10%
555	Control	Overall	93.68% ± 2.03%
108	Cog. impairment	Monosyllabic	78.70% ± 7.85%
109	Cog. impairment	Multisyllabic	91.67% ± 5.30%
217	Cog. impairment	Overall	85.19% ± 4.78%

*Note.* Cog. = cognitive.

Human annotators were provided with audio segments produced by FA of each selected word and were asked to identify whether the sound corresponded to the intended word in the transcript. Three responses were available: The sound contains most of the desired word (consistent with the “success” metric of Mahr et al.), the sound contains exactly the desired word fully correctly segmented, or the sound does not contain the desired word. Table 2 gives the results of human validation of the automatic alignment process. Overall alignment performance was worse for patients with cognitive impairment (*p* < .05). The difference was most marked for monosyllabic words, and it did not differ significantly between groups for the multisyllabic words. These results are comparable to those obtained by Mahr et al. (2021).

### Utterance-Level Diarization Accuracy

Diarization accuracy at the utterance level is based on the use of the start time of the first word in the utterance and the end time of the last word. This means that utterance-level diarization is guaranteed to be at least as accurate as word-level diarization accuracy and often higher because those inaccuracies that arise in the middle of the utterance do not affect utterance-level diarization accuracy.

### Adherence to CHAT Format

The pipeline is designed to produce a transcript that fully adheres to CHAT format, thereby permitting further profiling analysis. This adherence is checked in the sixth step of the pipeline. After MOR tagging, a final check uses the Chatter XML validator retrievable from https://talkbank.org/software/chatter.jar. Once the transcript passes this final check, it is in full compliance with the CHAT standard. All transcripts in our test set from the Delaware corpus passed this level of evaluation.

### Clean-Up Minimization

A basic goal of this research is to minimize the human transcriber's work required to produce an accurate, diarized, and tagged transcript for further LSA. Up to the sixth step of human correction, processing takes about 7 min for a 30-min audio sample with about 3 of the 7 min used in the speaker assignment step. At that point, relying on the CLAN editor's continuous playback function, the transcriber goes through the whole result of Batchalign to fix errors. For a 30-min recording of data of the type used in our current testing, this work will take between 60 and 90 min. This reduces total transcription time from about 10 hr to less than 2 hr. There are a variety of factors that will make these numbers vary markedly, including the quality of the audio, the nature of the interaction, the number of speaker overlaps, the accuracy of speakers' pronunciations, the language status of the participants, and the training level of the transcriber. When there are problems along these dimensions, transcriber time can increase markedly. If the sample comes from a speaker with marked phonological disorder, it may not be appropriate to use Batchalign at all. These issues are considered further in the Discussion section.

## Discussion

We have described a new suite of automated facilities for speech data recognition, segmentation, alignment, and analysis that can generate properly formatted CHAT transcripts with time alignments on the word and utterance levels. The Batchalign pipeline can be run automatically except for two segments that require human intervention. It allows clinicians and researchers to create CHAT transcripts automatically, providing access to additional facilities in CLAN for automatic morphosyntactic analysis and automatic profiling while minimizing the need for time-consuming manual transcription. Thus, use of Batchalign can markedly shorten the gap between data collection and data analysis.

An additional feature of Batchalign is its potential to address the issues of reliability and replicability (Munafò et al., 2017). Measures such as Cohen's kappa (Cohen, 1968) cannot be used to determine reliability between two human transcribers because words, unlike codes, are not nominal variables. Some transcribers may be more accurate than others, and the performance of a given transcriber can vary markedly across days and materials. Like human transcribers, the pipeline is not fully accurate. However, its accuracy in terms of WER, time boundaries, and utterance segmentation can be measured, evaluated, and replicated in a deterministic manner. Moreover, future advances in training sets and algorithms for speech technology can lead to continuous increases in precision and recall.

We are interested in providing versions of the pipeline that can work with other analysis systems, such as ELAN, SALT, and SUGAR, as mentioned earlier. Batchalign already includes format conversion programs for ELAN and Praat, and CLAN includes format conversion commands for SALT and SUGAR. These conversions can be made available as options. Some users may wish to analyze data with Python scripts. For this work, researchers can make use of the XML and JSON versions of CHAT files that can be created using the Chatter XML validator.

In this study, we focused on the E2E use of the Batchalign pipeline to create transcripts from raw audio. However, there is another entry point to the pipeline whereby existing transcriptions with rough human-annotated utterance-level alignments can be forced-aligned for word-level diarization and more accurate utterance-level diarization. Many of the existing transcripts in TalkBank have exactly this format because they were created by human transcribers using methods that aligned utterances with media in an approximate manner. Improving on the diarization of these transcripts involves running with the *-prealigned* switch that invokes only the use of MFA.

Despite its advantages, this method still faces at least six important limitations, as follows.

1. Accurate ASR requires high-quality audio input. Recordings made in noisy classrooms, testing rooms with fan noise, or home environments with poor acoustics will present challenges to ASR accuracy. Clever placement of digital recording devices and reduction of noise sources can alleviate some of these problems.
2. The recordings we have used thus far involve two-speaker interactions with only moderate amounts of overlap. Dealing with speaker overlap is currently a major challenge for ASR systems (Huang et al., 2022).
3. Our current use of the pipeline has focused on protocol interviews with adults. We expect that there will be greater problems involved in use of the pipeline with children, particularly very young children. However, our initial tests with school-age children in the Illinois International Stuttering Research Project (Yairi & Ambrose, 2005) corpus at https://fluency.talkbank.org/access/Password/IISRP.html and the MacWhinney longitudinal corpus of two siblings at https://childes.talkbank.org/access/Eng-NA/MacWhinney.html indicate that the pipeline performs well with both normal and moderately disordered child language samples.
4. There are also major challenges for ASR with adult speakers with disordered articulation or stuttering. There have been efforts to train corpora for adults who stutter (Lea et al., 2021) and adults with dysarthria (Kim et al., 2008). However, work along these lines is still in progress. In general, Batchalign would not be a good choice for work with samples from participants with phonological disorders. It would not work well with samples from people with fluent aphasia marked by frequent paraphasias. It would also not work well with people with global aphasia, apraxia of speech (Haley et al., 2012), or logopenic primary progressive aphasia (PPA; Keator et al., 2019). At the same time, our initial tests with other data types in TalkBank indicate that it can be used with children ages 4 years and older who have no clear phonological problems (Moyle et al., 2007). It can also be used with adults with dementia (Luz et al., 2021), TBI (Elbourn et al., 2023), RHD (Minga et al., 2022), some forms of PPA (Tippett et al., 2017), and Parkinson's disease. We are now using Batchalign on a regular basis when adding new, but not yet transcribed, data to the segments of TalkBank that include language from participants in these clinical groups.
5. Thus far, we have only used the pipeline with data from English. Our work with language from Australians with TBI has indicated that Rev AI does a good job with this dialect of English. How well it will work with other dialects is not yet clear. We have also used it successfully with assessments in Spanish for psychosis, but the absence of data for training tokenization for that language makes additional hand retokenization necessary. Outside of Spanish, we have no experience using the pipeline for yet other languages, although Rev AI includes recognizers for 36 languages.
6. Some projects may collect data that are subject to Health Insurance Portability and Accountability Act restrictions, which then further block the use of external services such as Rev AI. For such cases, we plan to make use of the open-source Whisper system from OpenAI (https://openai.com/blog/whisper; Baevski et al., 2020).

Given these limitations and gaps in testing, we would currently recommend that the pipeline be used primarily for transcription and analysis of two-party conversations between English-speaking adults who do not have marked articulatory or phonological problems. Future work will focus on quantifying and adjusting to these various limitations and maximizing the accuracy of the current system.

Another goal for future research will be the construction of baselines for model evaluation. Constructing these baselines will involve sampling from a wide variety of recording types and clinical types. A recent Association for Computational Linguistics workshop highlighted this as a general problem, particularly for the study of language disorders (Church et al., 2021). Stoppa et al. (2022) explain how the lack of a baseline data set for testing has restrained progress on the application of ML methods to the detection of the early onset of dementia. To correct for this problem, we (Luz et al., 2021) have established the Pitt corpus in DementiaBank as the baseline for algorithm testing through Interspeech challenges in 2020 (https://dementia.talkbank.org/ADReSS-2020/) and 2021 (https://dementia.talkbank.org/ADReSS-2021/). We hope to create similar methods, baseline corpora, and challenges for the study of ASR applications for language disorders.

## Data Availability Statement

The Batchalign software can be downloaded from https://github.com/talkbank. The data analyzed in this article come from AphasiaBank (https://aphasia.talkbank.org), TBI Bank (https://tbi.talkbank.org), and DementiaBank (https://dementia.talkbank.org) and are freely available to researchers, instructors, and clinicians after sending a request for access to macw@cmu.edu.

## Ethics Statement

Data for this work were retrieved from TalkBank repositories at https://talkbank.org. The development of TalkBank is supported by grants from the National Institutes of Health and National Science Foundation, and the system has received the Core Trust Seal as a trusted data repository (https://www.coretrustseal.org). TalkBank contains data contributed from projects that have been approved for data sharing by local institutional review boards. Contributors provide a statement indicating the nature of this review and any necessary restrictions on the use of the data.

## Author Contributions

**Houjun Liu:** Conceptualization (Lead), Data curation (Supporting), Formal analysis (Lead), Methodology (Equal), Project administration (Supporting), Resources (Equal), Software (Lead), Validation (Equal), Writing – original draft (Equal). **Brian MacWhinney:** Conceptualization (Supporting), Data curation (Lead), Formal analysis (Supporting), Funding acquisition (Lead), Methodology (Equal), Project administration (Lead), Resources (Equal), Software (Supporting), Supervision (Lead), Validation (Equal), Writing – original draft (Equal), Writing – review & editing (Lead). **Davida Fromm:** Data curation (Supporting), Funding acquisition (Supporting), Methodology (Supporting), Project administration (Supporting), Resources (Equal), Writing – original draft (Supporting). **Alyssa Lanzi:** Data curation (Supporting), Funding acquisition (Supporting), Methodology (Supporting), Project administration (Supporting), Resources (Equal), Writing – original draft (Supporting).
